# The development and validation of an LC-MS/MS method for the determination of a new anti-malarial compound (TK900D) in human whole blood and its application to pharmacokinetic studies in mice

**DOI:** 10.1186/1475-2875-13-42

**Published:** 2014-01-31

**Authors:** Efrem T Abay, Jan H van der Westhuizen, Kenneth J Swart, Liezl Gibhard, Matshawandile Tukulula, Kelly Chibale, Lubbe Wiesner

**Affiliations:** 1 Division of Clinical Pharmacology, Department of Medicine, University of Cape Town, Observatory, 7925 Cape Town, South Africa; 2 PAREXEL® International Clinical Research Organisation, Private Bag X09, Brandhof 9300, Bloemfontein, South Africa; 3 Department of Chemistry, University of the Free State, PO Box 339, Bloemfontein 9300, South Africa; 4 Department of Chemistry, University of Cape Town, Rondebosch 7701, Cape Town, South Africa; 5 Institute of Infectious Diseases and Molecular Medicine, University of Cape Town, Rondebosch 7701, Cape Town, South Africa

**Keywords:** Malaria, Drug development, Pharmacokinetics

## Abstract

**Background:**

Malaria is one of the most lethal and life-threatening killer infectious diseases in the world, and account for the deaths of more than half a million people annually. Despite the remarkable achievement made in preventing and eradicating malaria, it still remains a threat to the public health and a burden to the global economy due to the emergence of multiple-drug resistant malaria parasites. Therefore, the need to develop new anti-malarial drugs is crucial. The chemistry department at the University of Cape Town synthesized a number of new CQ-like derivatives (TK-series), and evaluated them for *in vitro* activity against both CQ-sensitive and -resistant *Plasmodium falciparum* strains, and for general cytotoxicity against a Chinese Hamster Ovarian (CHO) mammalian cell line. The lead compounds from the TK-series were selected for a comprehensive pharmacokinetic (PK) evaluation in a mouse model.

**Methods:**

A sensitive LC-MS/MS assay was developed for the quantitative determination of TK900D. Multiple reaction monitoring (MRM) in the positive ionization mode was used for detection. The analyte and the internal standard (TK900E) were isolated from blood samples by liquid-liquid extraction with ethyl acetate. Chromatographic separation was achieved with a Phenomenex® Kinetex C18 (100 × 2.0 mm id, 2.6 μm) analytical column, using a mixture of 0.1% formic acid and acetonitrile (50:50; v/v) as the mobile phase. The method was fully validated over concentrations that ranged from 3.910 to 1000 ng/ml, and used to evaluate the PK properties of the lead compounds in a mouse model.

**Results:**

The assay was robust, with deviation not exceeding 11% for the intra- and inter-run precision and accuracy. Extraction recovery was consistent and more than 60%. PK evaluation showed that TK900D and TK900E have moderate oral bioavailability of 30.8% and 25.9%, respectively. The apparent half-life ranged between 4 to 6 h for TK900D and 3.6 to 4 h for TK900E.

**Conclusion:**

The assay was sensitive and able to measure accurately low drug levels from a small sample volume (20 μl). PK evaluation showed that the oral bioavailability was moderate. Therefore, from a PK perspective, the compounds look promising and can be taken further in the drug development process.

## Background

Malaria, one of the world’s most serious and prevalent infectious diseases, has been and remains responsible for far more morbidity and mortality than most other diseases, especially in Africa. It has been estimated that in 2010 there were approximately 219 million cases of malaria that resulted in 660 000 deaths, 90% of which occurred in Africa [1]. Even though there is a tremendous increase in funding and intense momentum to reduce and/or eradicate malaria infections, the disease still remains a threat and an enormous burden on the global economy. This is due to the emergence of multiple-drug resistance of *Plasmodium falciparum,* the main cause of malaria infection in humans [1,2]. Therefore, the need to discover and develop new anti-malarial drugs is imperative.

**Figure 1:**
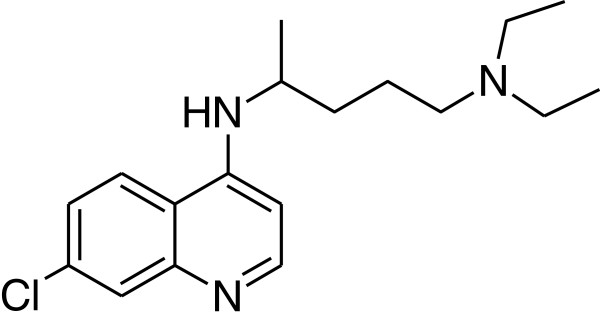
Chloroquine.

Chloroquine (CQ, Figure 1) was discovered by Hans Andersag and co-workers in 1934, but was ignored for a decade because it was considered toxic to humans. However, this notion changed when it was first introduced to clinical practice as a prophylactic treatment for malaria in 1947. Since then, and until the emergence of CQ-resistant *P. falciparum* strains, CQ was considered as the universal remedy for malaria and consequently a number of potent anti-malarial compounds were developed that were based on CQ core structure, *i.e.* the aminoquinoline nucleus [3]. The emergence of *P. falciparum* strains that were resistant to many drugs resulted in a serious limitation in existing anti-malarials; this necessitated the development of new anti-malarial drugs.

Several studies on the structure-activity relationship of the aminoquinolines were undertaken in order to improve their activity against drug-resistant *P. falciparum* strains. Ridley *et al*. [4] and De *et al*. [5] observed that shortening of the CQ alkyl side-chain length to 2 – 3 carbon atoms, and lengthening it to 10 – 12 carbon atoms resulted in compounds that were active against CQ-resistant *P. falciparum* strains. Stocks *et al*. [6] reported that CQ derivatives in which the diethyl amino function of the CQ’s side-chain was replaced by metabolically more stable groups (such as *tert*-butyl, piperidyl or pyrrolidino) led to a significant increase in anti-malarial activity against the CQ-resistant strains. According to Iwaniuk *et al*. [7] modifying the length and basicity of the CQ side chain, in particular the 4-amino-7-chloroquinolines, with a linear side chain that consists of two aliphatic tertiary amino functions, enhanced the anti-malarial activity against both CQ-resistant and -sensitive strains.

**Figure 2:**
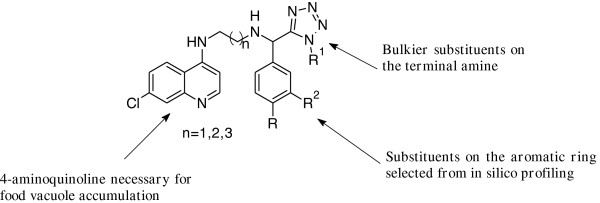
Rationale design for chloroquine-based target compounds.

Thus encouraged by the aforementioned findings, the Department of Chemistry at the University of Cape Town designed and synthesized a number of new CQ-like derivatives [8]. The design focused mainly on avoiding the commonly observed metabolic *N*-dealkylation in CQ-derivatives by incorporating bulkier substituents such as the aromatic and tetrazole rings, while varying the length of the alkyl side-chain (Figure 2).

All the synthesized CQ-like derivatives were evaluated *in vitro* for potency against both CQ-sensitive (3D7) and CQ-resistant (K1 and W2) strains of *P. falciparum.* The *in vitro* antiplasmodial activity IC_50_ values for TK900D were 0.0004, 0.0082, and 0.0305 μM against 3D7, K1 and W2 strains respectively. Compared to CQ, TK900D was less active (CQ IC_50_ 0.0002 μM) against the CQ-sensitive strain but significantly more active against the CQ-resistant strains, K1 and W2 (IC_50_.values of CQ 0.036 and 0.0591 μM, respectively).

Additionally, TK900D was found to be highly selective towards Plasmodium infection based on the results obtained from *in vitro* cytotoxicity test against a CHO mammalian cell line, using the 3-(4, 5-dimethylthiazol-2-yl)-2, 5-diphenyltetrazoliumbromide (MTT) assay (IC_50_ value of 10.5 μM). Thus, compound TK900D and its related compound TK900E were selected as the lead compounds for comprehensive PK evaluation as the evaluation of the PK properties of the lead compounds is a prerequisite for lead prioritization in the drug discovery and development process.

In this paper, the development and validation of sensitive and selective LC-MS/MS assay methods that can accurately measure drug levels from a small extraction volume (20 μl) of mice blood, and its application to the evaluation of the PK properties of the compounds in a mouse model is presented.

## Methods

### Chemicals and reagents

**Figure 3:**
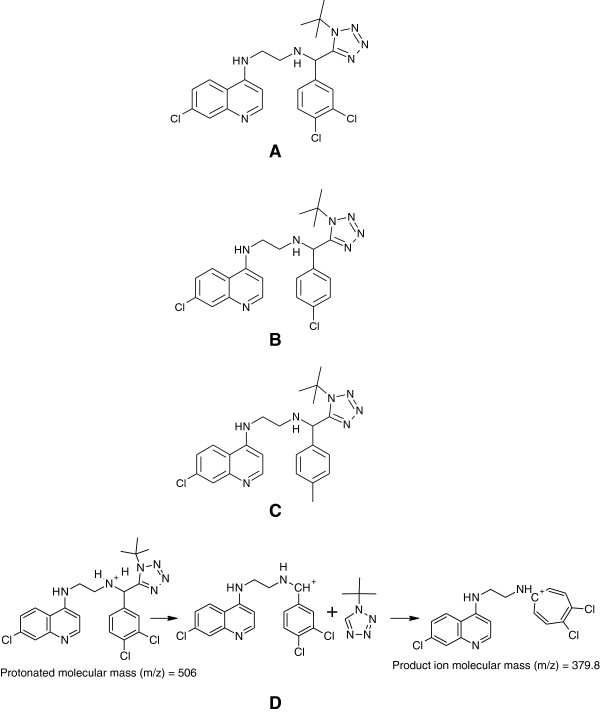
Structures of (A) TK900D; (B) TK900E (C) TK900C and (D) fragmentation pattern of TK900D.

TK900D (C_23_H_24_Cl_3_N_7_, MW = 504.85; Figure 3A) and TK900E (C_23_H_25_Cl_2_N_7_, MW = 470.40; Figure 3B) were synthesized and their HPLC purity was determined to be > 99%. All chemicals and reagents used in this study were of analytical grade or ACS (American Chemical Society). Ammonium formate (97% pure) was purchased from Sigma-Aldrich Gmbh (Steinheim, Germany), formic acid (98 – 100%) was purchased from Merck KGaA (Darmstadt, Germany), acetonitrile, ethyl acetate and methanol (all of high-purity grade) were purchased from Honeywell, Burdick & Jackson (Muskegon, MI 49442, USA). Water used to prepare solutions was purified by a Millipore Elix 10 reverse osmosis and Milli-Q® (Millipore, USA) Gradient A 10 polishing system.

### Chromatography

HPLC analysis was performed with an Agilent 1200 infinity series quaternary pump which delivered the mobile phase at a flow rate of 250 μl/min, combined with an Agilent 1200 infinity series auto-sampler, degasser and column compartment. The auto-sampler was equipped with a 96-well plate and was used to inject 5 μl samples onto the HPLC column. The Agilent cooling device was set at 5°C. Chromatography was performed on a Phenomenex® Kinetex C18 (100 × 2.0 mm id, 2.6 μm) analytical column fitted with a Phenomenex® Security Guard™ System containing a C18 (4 × 3 mm) pre-column. The column was kept at 30°C with an Agilent 1200 infinity series column compartment.

### Detection

Analysis was performed on an AB SCIEX API 3200 triple quadrupole mass spectrometer (AB SCIEX, Toronto, Canada) equipped with an electrospray ionization (ESI) source operated at 550°C and set in the positive ion mode for ion production. Transition of the protonated precursor ions m/z 506 and m/z 472, to the product ions m/z 380 and m/z 346 for TK900D and the internal standard (TK900E), respectively, were monitored at unit resolution in the multiple reaction monitoring (MRM) mode with a dwell time of 200 ms per transition. The curtain, nebulizer, turbo, and collision gases were set at 20, 35, 35 and 3 psi, respectively, while the ion spray voltage and the source temperature were set at 2000 V and 550°C, respectively. The declustering potential, collision energy, entrance potential, and collision cell exit potential were optimized at 65, 35, 4, and 6 V respectively for TK900D; and 50, 33, 3, and 6 V respectively for the internal standard. The instrument was interfaced to a work station running Analyst™ version 1.5.2 software and all data generated was captured and stored on the work station’s hard disc drive.

### Preparation of calibration standards and quality control samples

Human whole blood containing lithium heparin as anticoagulant was donated by volunteers at the clinical division of PAREXEL International (South Africa) Bloemfontein. A stock solution of TK900D at a concentration of 95.39 μg/ml was prepared by dissolving 1.021 mg of TK900D in 10.703 ml of methanol (i.e. equivalent to 8.466 g of methanol). A pool of human blood (5 g) was spiked with 50 μl of TK900D stock solution to obtain a calibration standard at upper limit of quantification (ULOQ) of 1000 ng/ml, which was serially diluted with blank blood down to 3.910 ng/ml, the lower limit of quantification (LLOQ). A different stock solution was prepared and the same methodology was followed to prepare the quality control standards, which ranged from 3.910 to 800.0 ng/ml. Samples were aliquoted (40 μl) in a 1.5 ml polypropylene microfuge tubes and stored at approximately -80°C.

### Sample preparation

Blood samples were completely thawed unassisted at room temperature and briefly vortexed. Fifty microlitres of a 20 mM ammonium formate buffer (pH 5.5) were added to microfuge extraction tubes, 20 μl of the blood sample was added, followed by the internal standard (100 μl of 100 ng/ml TK900E in water). After a brief vortex mixing of the sample, 1 ml of ethyl acetate was added and vortexed for two minutes followed by centrifugation at 2000 g for five minutes at 4°C. The aqueous phase was frozen in an alcohol freezing bath at ~ -20°C, and the organic phase transferred into clean polypropylene tubes and evaporated to dryness (~ 40°C) under a gentle stream of nitrogen gas. The residue was reconstituted with 200 μl of a 0.1% formic acid and acetonitrile solution (50:50; v/v) and vortexed for 40 seconds. Extracts were transferred into a 96-well plate and 5 μl of the sample was injected onto the HPLC column.

### Method validation

The method was validated according to the bioanalytical method validation guidelines of the US Food and Drug Administration [9] and the European Medicines Agency [10] by analysing an appropriately prepared calibration, and quality control standards in three consecutive batches to demonstrate acceptable intra- and inter-batch accuracy and precision over the desired range of concentration. Quantification models based on peak areas and peak area ratios were assessed to determine which model performed the best for the statistical analysis of the validation batches. A batch included all the calibration standards in duplicate from 3.910 to 1000 ng/ml (LLOQ to ULOQ), seven quality control standard levels spanning the concentration range from 3.910 (LLOQ) to 800.0 ng/ml (QC high) in replicates of six, six blanks, two double blanks and three system performance verification samples (SPVS) at the beginning, middle and end of the batches.

### Assay specificity

Blank human blood samples obtained from ten different sources were tested for any visible interference.

### Matrix effect

In order to evaluate the matrix effect on the ionization of the analytes, blank human blood samples obtained from ten different sources were extracted and spiked to high (800.0 ng/ml) and low (10.01 ng/ml) concentrations of the analyte and one concentration of the internal standard (100.0 ng/ml). These samples were injected together with samples containing no matrix components.

### Linearity

Standard curves (n = 3) of nine different concentration levels of TK900D (3.910-1000 ng/ml), including blanks (n = 6) to control the carry-over and the presence of any interferences, double blanks (n = 2) to ensure that the internal standard did not interfere with the quantification of the analyte, and three system performance verification samples to evaluate the instrument response over the total run time, were extracted and assayed.

### Inter-batch accuracy (% Nom) and precision (% CV)

The inter-batch accuracy and precision of the assay procedure were assessed by calculating the accuracy and precision statistics of the seven levels of quality control standards (n = 6 per batch) over all three validation runs.

### Extraction efficiency

Absolute recovery of the extraction procedure was assessed by comparing the responses of spiked extracts with the quality control standards (n = 6) at high (800.0 ng/ml), medium (160.1 ng/ml) and low concentrations (10.01 ng/ml) of TK900D and at one concentration of the internal standard (100.0 ng/ml) in whole blood.

### Stability

#### Stock solution stability

The stability of TK900D and TK900E in methanol was evaluated at room temperature, ~ 5°C and ~ -20°C. Stock solutions with concentrations of 100.0 μg/ml of TK900D and the internal standard were prepared in methanol. Three aliquots of each of the stock solutions were kept at room temperature, ~ 5ºC, and ~ –20°C, respectively, for eight days. After diluting the stored stock solutions in injection solvent to a 100.0 ng/ml, the stability of TK900D and that of the internal standard were assessed by comparing the peak areas obtained from the stored stock solutions with peak areas of the freshly prepared stock solutions. For stock solution results to be acceptable the percentage reference value should not exceed 15%.

#### Long-term stability

For the determination of long-term stability in human whole blood, TK900D spiked quality control samples at 800.0 ng/ml and 10.01 ng/ml were stored at ~ -80°C for 181 days (long enough to cover the time period elapsed from the first day of sample collection to the final sample analysis). These samples were thawed on the day of testing and run together with freshly prepared calibration standards and quality controls and the values were calculated from the resulting calibration curve obtained from the calibration standards.

#### Freeze and thaw stability

Quality control blood samples at high and low concentration, 800.0 and 10.01 ng/ml respectively, of TK900D stored frozen at ~ -80°C were allowed to thaw completely unassisted at room temperature and then refrozen for 12 to 24 hours. After three such freeze-thaw cycles the samples were assayed in the third validation run and the measured concentrations were compared with the nominal concentrations of these samples.

#### Short-term (on-bench) stability

Quality control samples at high and low concentrations (800.0 ng/ml and 10.01 ng/ml, respectively) of TK900D were thawed completely unassisted at room temperature and kept on bench for a period of time required to prepare/extract the samples (~ 4 to 6 h.). The samples were assayed in one of the validation batches. The measured concentrations were compared with the nominal concentrations of these samples.

#### On-instrument stability

In order to assess the stability of the analytes while awaiting injection on instrument, on-instrument stability (OIS) was assessed for the period of time that the extracted samples were expected to stay on-instrument during the batch run-time (~ 9 h). Quality control samples at high and low concentrations (800.0 ng/ml and 10.01 ng/ml, respectively), were extracted in replicates of six and injected at the beginning and end of the run (i.e. six QC-high and six QC-low at the beginning of the run and another set of six QC-high and QC-low at the end of the run bracketed with quality control samples). The mean measured concentration of the OIS-samples (injected at the end of the run) and OIS-reference samples (injected at the beginning of the run) were compared: in order to be acceptable, their percentage difference should be within ± 15%.

#### Cross validation of human and mouse blood

According to the EMA Guidelines on Bio-analytical Method Validation, 2012 [9], differences in sample preparation, different matrices or the use of another analytical method may result in different outcomes between the study sites. If possible, a cross-validation should be performed in advance of the study samples’ analysis. For cross-validation, the same set of QC samples or study samples should be analysed by different analytical methods or by means of the same method using different matrices. For QC samples, the obtained mean accuracy using the two different matrices or different methods should be within 15% and may be wider, if justified.

The efficacy and bioavailability studies were performed in a mouse model [8], but due to the scarcity of mouse blood, the method development and validation of the LC-MS/MS assay were performed using human whole blood. A cross validation by analysing the blood of mice spiked with analytes at LLOQ, low, medium and high concentration levels (3.909, 10.01, 160.1 and 800.0 ng/ml) in six fold against calibration standards and quality controls prepared in human whole blood was performed to check that the validation parameters will generate the same results (± 15% variation) in both matrices.

## Results and discussion

### LC-MS/MS optimization

**Figure 4:**
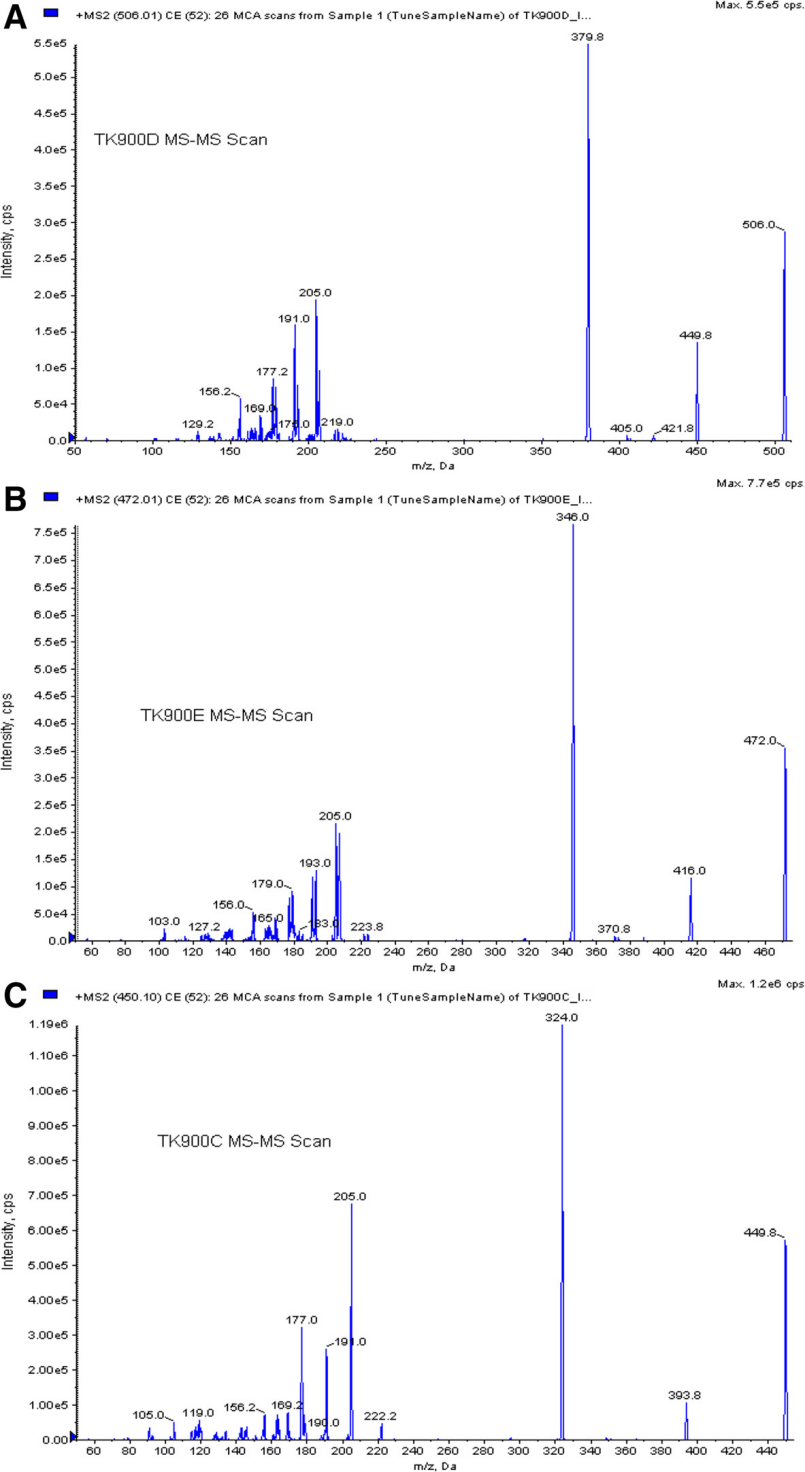
MS/MS spectrum of (A) TK900D; (B) TK900E (C) TK900C.

Due to the presence of a number of amine groups in the structures of TK900D and the IS an ESI in the positive ionization mode was selected for ion production. After collision-induced dissociation, the most abundant and stable product ions were at *m/z* 379.8 for TK900D and at *m/z* 346.0 for the IS (Figure 4). Thus, the MRM transitions of *m/z* 506 → 380 and *m/z* 472 → 346 were selected for TK900D and the IS respectively for the quantitative analysis. The mono-isotopic masses of TK900D and TK900E are 503.1159 and 469.1548, respectively. As a result, the masses of their protonated molecular ions were supposed to be 504 and 470 but instead, 506 and 472 were obtained during the setting up of the acquisition methods. During Q1-scan, the infusion mass spectrum of TK900E shows that the mass of the protonated molecular ion with the most intense spectrum belongs to 470, followed by 472 and 471. However, during compound optimization and the fragmentation process, the instrument selected the protonated molecular ion with a mass of 472, as presented in Figure 4B (MS/MS spectra of TK900E). This is due to the presence of multiple chlorine atoms in both molecules which has an influence on the multiplicity of the isotope peaks [11]. The presence of more than one chlorine atom in a molecule makes the multiplicity of the isotope peaks more complex and the x + 2 peak becomes more intense (x stands for the mass of the protonated molecular ion with the most abundant chlorine isotope, ^35^Cl, thus x + 2 represents the mass of the protonated molecular ion with ^37^Cl).

Six types of column, namely Discovery C18 (2.1 mm × 150 mm, 5 μm), Discovery C8 (2.1 mm × 150 mm, 5 μm), Discovery Cyano (2.1 mm × 150 mm, 5 μm), Kinetex C18 (2.0 mm × 100 mm, 2.6 μm), Luna C18 (2.0 mm × 150 mm, 5 μm), and Luna Phenyl Hexyl (2.0 mm × 150 mm, 5 μm) were tested for chromatographic parameters, such as retention time variability, peak shape, resolution, etc. – and the best result was obtained with Kinetex C18, followed by Discovery C18 and Luna C18 as a second and third choice, respectively.

For the optimal selection of the mobile phase, various mixtures of solvents such as methanol, acetonitrile, and methanol-acetonitrile (1:1, v/v) with volatile buffers such as 0.1 to 0.5% formic acid and 20 mM ammonium formate were tested to establish the efficiency of their MS ionization, the variability of their retention time, and the shape of the peak obtained. The best result was attained with 0.1% formic acid-acetonitrile (50:50, v/v) as the mobile phase at a flow rate of 250 μl/min.

Optimization of the injection solution was also done by testing 0.1% formic acid, acetonitrile, and the mobile phase as an injection solution. The mobile phase was found to be the best injection solution which resulted in the best shape of chromatographic peak with higher intensity (best MS ionization) and a stable retention time.

**Figure 5:**
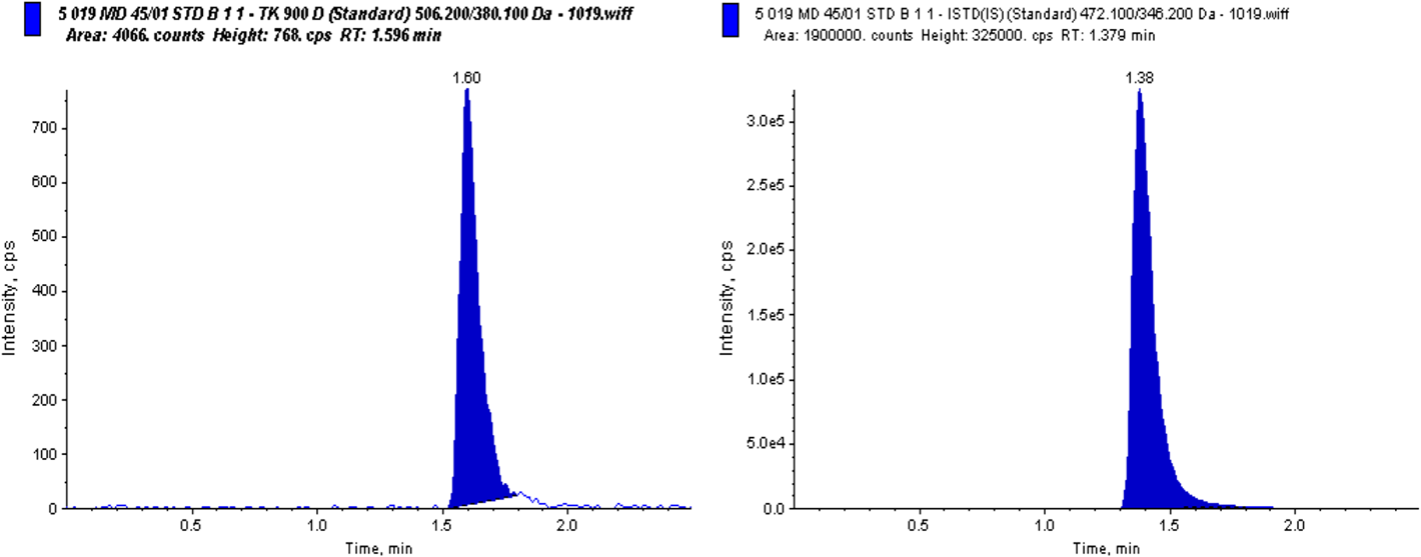
Representative chromatogram of TK900D at LLOQ.

The total run time was 2.5 minutes per sample. A representative chromatogram of a calibration standard at LLOQ is presented in Figure 5.

### Sample preparation

Blood samples were processed by protein precipitation with ice-cold acetonitrile and LLE with different organic solvents, such as hexane-isoamyl alcohol (98:2; v/v), diethyl ether, ethyl acetate, hexane-ethyl acetate (60:40; v/v) and tert-butyl methyl ether (TBME). In addition to the higher extraction recovery due to the cleaner extracts obtained, LLE was preferred to protein precipitation. Among the different organic solvents tested for sample preparation, the best extraction efficiency (recovery) was obtained with ethyl acetate. Extraction with and without buffers at various pH values, were tested, and the best results were obtained with a 20 mM ammonium formate buffer at pH 5.5.

### Method validation

#### Assay specificity

**Figure 6:**
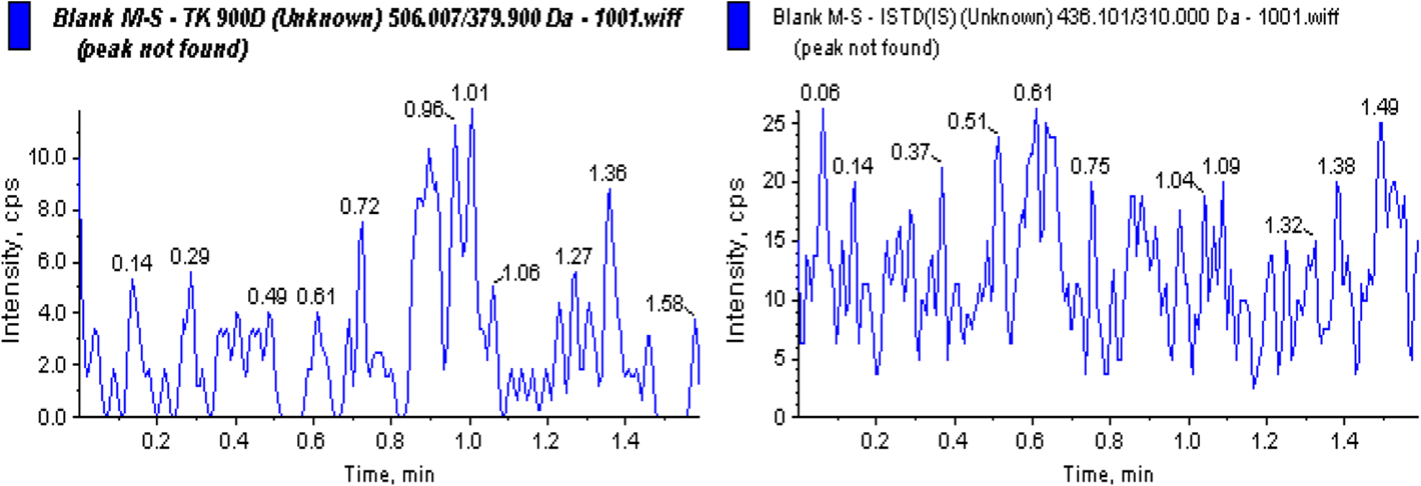
Representative chromatogram of TK900D blank human whole blood extract.

Blank human blood samples obtained from ten different sources were tested for any visible interference. A representative chromatogram of a blank extract, as shown in Figure 6, indicates that there was no interference, i.e. no endogenous peaks at or near the retention time of the analyte or the internal standard.

#### Linearity and LLOQ

The quantification of TK900D over the entire range, 3.910-1000 ng/ml was performed based on peak area ratios using a Wagner calibration curve (ln(y) = a(ln(x))^2^ + b(ln(x)) + c) and r^2^ of 0.9991. The cumulative results of three representative standard curves for TK900D are presented in Table 1.

**Table 1:** Cumulative statistics of TK900D calibration standards and quality control samples

**Parameters**	**Calibration standards and nominal concentrations (ng/ml)**								
	**STD B**	**STD C**	**STD D**	**STD E**	**STD F**	**STD G**	**STD H**	**STD I**	**STD J**
	**3.910**	**7.821**	**15.64**	**31.28**	**62.57**	**125.0**	**250.0**	**500.2**	**1000**
**Mean**	4.051	7.524	15.48	30.94	64.10	126.6	251.7	496.6	996.3
**% Nom**	103.6	96.2	99.0	98.9	102.5	101.3	100.7	99.3	99.6
**% CV**	3.4	4.3	1.7	3.9	2.2	1.9	0.6	0.9	0.9
**% Bias**	3.6	-3.8	-1.0	-1.1	2.5	1.3	0.7	-0.7	-0.4
**N**	6	6	6	6	6	6	6	6	6
**Parameters**	**Quality control samples and nominal concentration (ng/ml)**								
	**QC A**	**QC B**	**QC C**	**QC D**	**QC E**	**QC F**	**QC G**	**QC H DIL**	
	**3.909**	**10.01**	**20.01**	**60.03**	**160.1**	**400.2**	**800.0**	**1600**	
	**LLOQ**	**Low**	**----**	**----**	**Medium**	**----**	**High**	**Dilution**	
**Mean**	3.815	10.12	21.13	63.42	177.5	436.2	840.9	1673	
**% Nom**	97.6	101.1	105.6	105.7	110.9	109.0	105.1	104.6	
**% CV**	10.8	5.3	4.5	5.4	5.7	7.1	8.3	5.1	
**% Bias**	-2.4	1.1	5.6	5.7	10.9	9.0	5.1	4.6	
**N**	18	18	18	18	18	18	18	6	

QCH DIL was used to establish the dilution linearity of the method.

#### Precision and accuracy

The within- and between-batch accuracy (% Nom) and precision (% CV) of the assay procedure were assessed by calculating the accuracy and precision statistics of the 7 levels of quality control standards (n = 6 per batch) over all three validation runs, as presented in Table 1. The deviation is within ± 15% of the nominal value at all the concentration levels. This indicates an acceptable accuracy and precision.

#### Extraction efficiency

The extraction recovery determined for TK900D was consistent and repeatable. The results are presented in Table 2.

**Table 2:** Absolute recovery, using response factor

**Sample**	**Analyte conc. (ng/ml)**	**Mean of peak areas**		**Absolute recovery (%)**	**CV (%)**
		**After extraction**	**Theoretical values**		
**High conc.**	800.0	825850	1120664	73.7	4.3
**Medium conc.**	160.1	169317	260280	65.1	4.5
**Low conc.**	10.01	10482	14370	72.9	8.9
**Mean**				70.6	5.9
**ISTD**	100.0	418683	543089	77.1	2.8

**N.B.:** The concentration of the ISTD was same at high, medium and low concentration levels.

#### Stability assessment

A summary of the stability assessment is presented in Table 3. This includes the analyte stability in stock solution, freeze-thaw stability, on-bench stability, long-term stability, and on-instrument stability. All the results showed that the analyte was stable under the conditions in which the stability assessment was performed, i.e.

•Both TK900D and the internal standard were stable in methanol at all the storage temperatures (at room temperature, 5°C, and -20°C);

•TK900D was stable in human whole blood for 181 days when stored at -80°C;

•TK900D was stable for at least three freeze-thaw cycles;

•TK900D was stable for ~ 12 h when left on-bench at room temperature; and

•Both TK900D and the internal standard were stable for ~ 8.2 h on-instrument.

**Table 3:** Stability assessment

**Stability**	**Analyte code**		**Mean analyte peak area (n = 6)**			
			**Room temperature**	**~ 5°C**	**~ -20°C**	**Fresh (reference)**
Analyte stock solution stability in methanol	**TK900D**	**Peak area**	813083	800550	762900	760700
		**% Reference**	106.9	105.2	100.3	N/A
		**% CV**	2.9	1.4	2.4	1.8
	**TK900E**	**Peak area**	876300	881567	836667	852133
		**% Reference**	102.8	103.5	98.2	N/A
		**% CV**	1.9	2.8	2.2	2.9
**Stability**			**TK900D Nominal concentration (ng/ml)**			
			**High (800.0)**		**Low (10.01)**	
**Long term**	**Mean**		805.7		9.598	
	**% CV**		6.9		11.9	
	**% Bias**		0.7		-4.0	
**Freeze and thaw**	**Mean**		852.7		10.87	
	**% CV**		5.8		8.9	
	**% Bias**		6.6		8.6	
**On bench**	**Mean**		866.0		10.53	
	**% CV**		3.4		7.5	
	**% Bias**		8.3		5.2	
**OIS**	**Mean**		806.9		10.46	
	**% CV**		0.6		1.4	
	**% Bias**		0.9		4.5	

All results are mean of n = 6.

#### Cross validation

No significant differences were found between the samples prepared in human blood and in blood from mice. This indicated that human blood could be used to prepare calibration standards and quality control samples. The results are presented in Table 4.

**Table 4:** Cross validation result summary for TK900D

**Species**	**Human**	**Mouse**	**Human**	**Mouse**	**Human**	**Mouse**	**Human**	**Mouse**
**Nominal conc. (ng/ml)**	**800.0**	**800.0**	**160.0**	**160.0**	**10.00**	**10.00**	**3.906**	**3.906**
**Mean (n = 6)**	809.2	899.3	160.8	185.7	9.889	10.66	3.912	3.946
**% CV**	7.5	5.4	8.2	5.6	9.1	12.2	9.4	11.9
**% Bias**	1.2	12.4	0.5	16.1	-1.1	6.6	0.2	1.0

#### Matrix effect

It has been noted that co-eluting, undetected endogenous matrix components may affect the ion intensity of the analyte and metabolite and adversely affect the reproducibility and accuracy of the LC-MS/MS [12]. In order to determine whether this effect (called the matrix effect) is present or not, normal blank human blood from 10 different sources was extracted, dried and reconstituted using solutions of high (800.0 ng/ml) and low (10.01 ng/ml) concentrations of the analyte and at one concentration of the internal standard (100.0 ng/ml). These samples were injected together with samples prepared in the reconstituted solution at the same concentrations, containing no matrix components.

The matrix effect is quantitatively measured by calculating the Internal Standard-Normalized Matrix Factor (IS-MF), which is the Peak Area Ratio in the Presence of Matrix Ions for each blood sample divided by the mean of the Peak Area Ratio in the Absence of Matrix Ions.

A matrix factor (MF) of one signifies no matrix effect, while a value of less than one suggests the suppression of ionization. A value that is greater than one signifies ionization enhancement [13]. An absolute Internal Standard-Normalized MF of one is not required for a reliable analytical assay. However, the variability (% CV) in matrix factors should be less than or equal to 15% to ensure reproducibility of the analysis.

The internal standard normalized matrix factor as calculated for this particular paper showed no significant ion suppression or enhancement at high and low concentrations of TK900D. The variability (% CV) was 2.6% and 2.8% at 800.0 ng/ml and 10.01 ng/ml, respectively, which indicates that sample analysis was reproducible.

### Pharmacokinetic evaluation of TK900D

Snapshot pharmacokinetic evaluations were performed on a number of analogues from the TK-series anti-malarial compounds. TK900D showed to be one of the most promising compounds from a pharmacokinetic perspective, and was selected for comprehensive pharmacokinetic evaluation. The test compound dissolved in a 20 mM Sodium acetate buffer (pH 4.0): Ethanol: PEG400 (70:5:25; v/v/) drug vehicle was administered orally to healthy C57/BL6 mice (n = 5) at doses of 40 and 20 mg/kg, and intravenously at doses of 5 and 2.5 mg/kg. Blood samples were collected at predetermined sampling times (except for the first sampling time, i.e. 5 minutes after dosing for the IV group and 10 minutes for the oral group, the sampling times were 0.5,1, 3, 5, 7, 12 and 24 h after dosing) by bleeding the tip of the tail, and analysed using a validated LC-MS/MS assay. Back-calculated concentrations of the blood samples were obtained from a standard regression curve with nine concentration levels (3.910 to 1000 ng/ml). Concentration *vs.* time profiles were constructed and the data analysed with Summit PK software to obtain the pharmacokinetic parameters.

**Figure 7:**
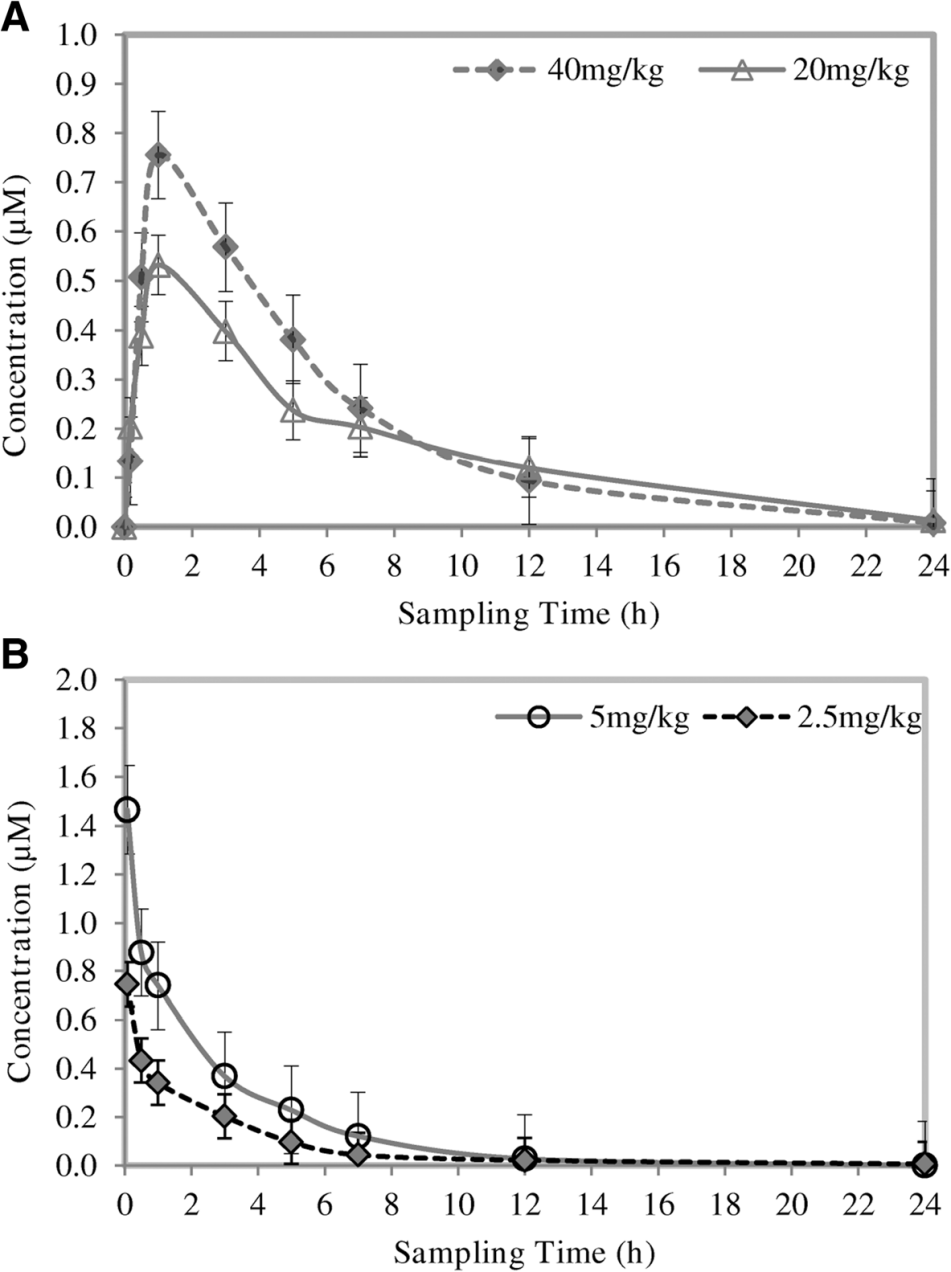
**Mean blood concentration *vs*. time profiles of TK900D following the administration of (A) 40 and 20 mg/kg TK900D orally and (B) 5 and 2.5 mg/kg TK900D intravenously to healthy male C57BL/6 mice (n = 5).**

The pharmacokinetic parameters are presented in Table 5 and the blood drug concentration *vs.* time profiles (mean of n = 5) are presented in Figure 7.

**Table 5:** Pharmacokinetic parameters for TK900D and TK900E in male C57/BL6 mice

**Parameters**	**TK900D**				**TK900E**			
	**Oral**^**a**^		**IV**^**a**^		**Oral**^**a**^		**IV**^**a**^	
**Nominal dose (mg/kg)**	40	20	5.0	2.5	40	20	5.0	2.5
**Apparent t**_**1/2 **_**(h)**	3.9	6.0	2.3	1.9	4.0	3.6	2.5	1.6
**Blood CL**_**total **_**(ml/min/kg)**	—^b^	—^b^	44.8	48.9	—^b^	—^b^	51.0	51.2
**Vd (l/kg)**	—^b^	—^b^	8.9	7.9	—^b^	—^b^	10.3	7.0
**Vss (l/kg)**	—^b^	—^b^	9.1	8.7	—^b^	—^b^	12.6	6.5
**C**_**max **_**(μM)**	0.79	0.54	—^b^	—^b^	2.81	0.94	—^b^	—^b^
**T**_**max **_**(h)**	1.4	1.4	—^b^	—^b^	1.0	0.8	—^b^	—^b^
**AUC**_**0 - ∞ **_**(min. μmol/l)**	287	256	222	104	541	222	221	107
**Bioavailability (%)**	16.2	30.8	—^b^	—^b^	30.6	25.9	—^b^	—^b^

^a^Values are the mean of 5 animals, ^b^Empty cells indicate that the value was measured or was not relevant.

The apparent half-life for TK900D ranged from 2 to 6 h. The volume of distribution was high (8.9 l/kg at 5.0 mg/kg, and 7.9 l/kg at 2.5 mg/kg doses) and the blood clearance moderate (44.8 ml/min/kg at 5.0 mg/kg, and 48.9 at 2.5 mg/kg doses). The mean blood drug concentrations were 0.79 μM and 0.54 μM and the AUC was 287 and 256 min.μmol/l for the high and low doses respectively. One would expect that by doubling the dose the C_max_ and AUC would increase significantly, but this was not observed – possibly indicating that the rate of solubility or dissolution limited the absorption at higher doses. The oral bioavailability of the drug in the groups that received relatively high doses (oral at 40 mg/kg, and IV at 5 mg/kg) was 16.2%, and the oral bioavailability of the drug in the groups that had relatively low doses (oral at 20 mg/kg, and IV at 2.5 mg/kg) was 30.8%.

According to the MMV (Medicines for Malaria Venture) compound progression criteria of August 2008 [14], a compound with oral bioavailability > 20% in rat after oral dosing is considered as a development candidate. Therefore, the oral bioavailability of TK900D in a mouse model looks promising.

### Pharmacokinetic evaluation of TK900E

**Figure 8:**
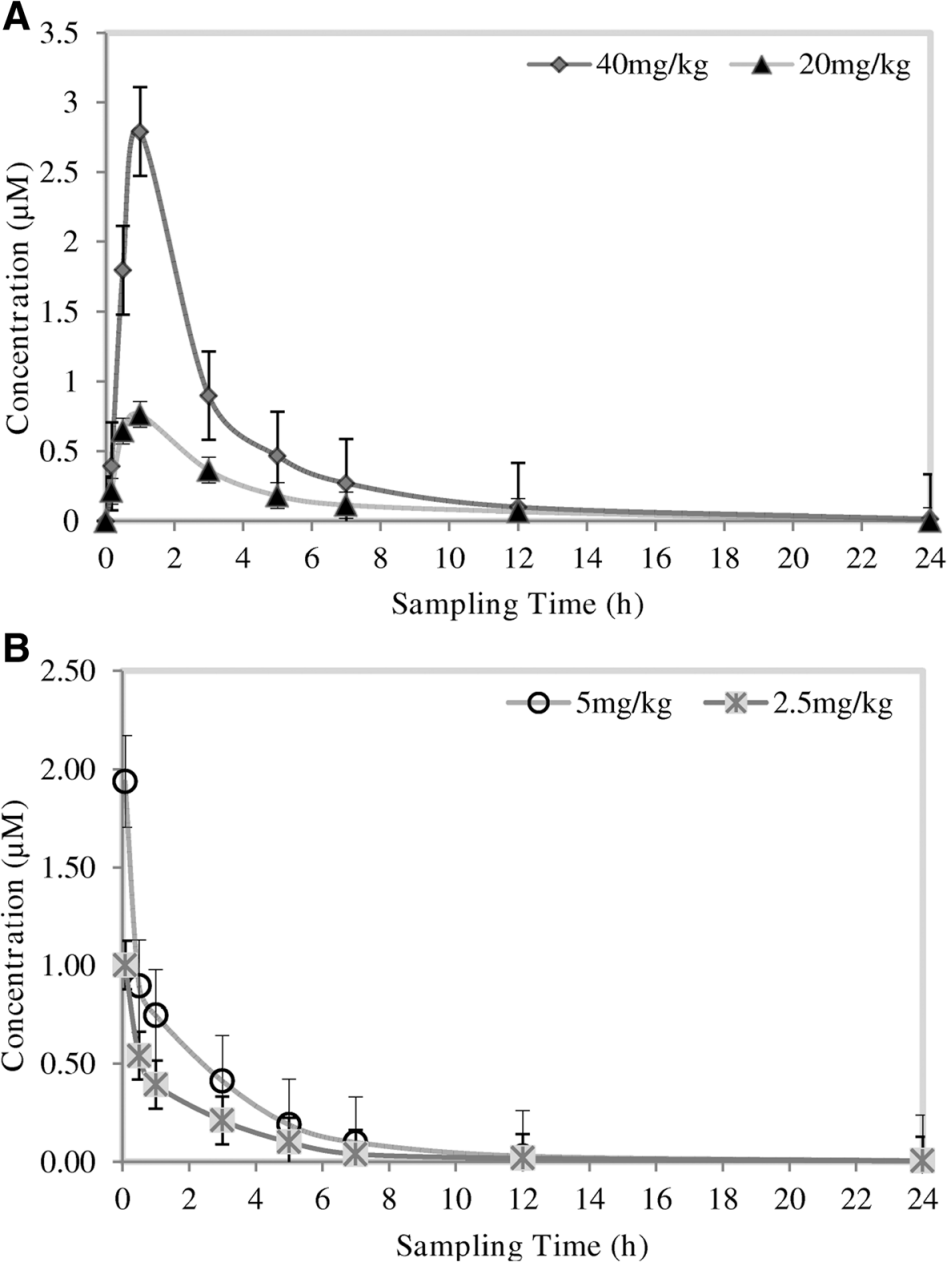
**Mean blood concentration *vs*. time profiles of TK900E following the administration of (A) 40 and 20 mg/kg TK900E orally and (B) 5 and 2.5 mg/kg TK900E intravenously to healthy male C57BL/6 mice (n = 5).**

TK900E, another CQ-like derivative in this chemical series, was evaluated for its PK properties in a mouse model. The *in vitro* IC_50_ values in both the CQ-sensitive (3D7) and -resistant (K1 & W2) *P. falciparum* strains were 0.002, 0.001 and 0.0255 μM, respectively. Thus, another LC-MS/MS method using the same LC conditions and extraction procedure as for TK900D, was developed and fully validated for TK900E, using TK900C (Figure 3C) as an internal standard (mass spectrum of TK900C is presented in Figure 4C). The validated method was used to evaluate the pharmacokinetic properties of TK900E in a mouse model and the results are presented in Table 5. The blood drug concentration *vs*. time profiles (mean of n = 5) data is presented in Figure 8.

The apparent half-life for TK900E ranged between 1.6 to 4 h. The volume of distribution was high (10.3 l/kg at 5.0 mg/kg, and 7.0 l/kg at 2.5 mg/kg doses) and the blood clearance moderate (51.0 ml/min/kg at 5.0 mg/kg, and 51.2 at 2.5 mg/kg doses).

The mean blood drug concentrations were 2.81 μM and 0.94 μM, and the AUC was 541 and 222 min.μmol/l for the high and low doses, respectively, indicating a dose-dependent relationship (doubling the dose nearly doubles the response in concentration and AUC).

The oral bioavailability of the relatively high dose groups (oral at 40 mg/kg, and IV at 5 mg/kg) was 30.6%, and the oral bioavailability at the relatively low dose groups (oral at 20 mg/kg, and IV at 2.5 mg/kg) was 25.9%.

## Conclusion

Robust LC-MS/MS methods were developed and validated for the quantification of TK900D and TK900E in blood, using a very small extraction volume (20 μl).

The reported method offers an advantage of rapid and simple liquid-liquid extraction, together with a short chromatographic run time. This makes the method suitable for the analysis of large sample batches without any loss in instrument performance. The signal-to-noise ratios (S/N) at the pre-set LLOQ value of 3.910 ng/ml, were 30 and 20 for TK900D and TK900E respectively. The S/N ratio indicates that the methods were highly sensitive; even though a small volume of extraction (20 μl) was used. The methods were successfully used to evaluate the pharmacokinetic parameters of TK900D and TK900E in a mouse model.

## Abbreviations

ACS: American chemical society; AUC: Area under the curve; CHO: Chinese hamster ovarian; Cmax: Maximum concentration; CQ: Chloroquine; CV: Coefficient of variation; EMA: European Medicines Agency; FDA: Food and Drug Administration; HPLC: High performance liquid chromatography; IC50: 50% inhibitory concentration; IS-MF: Internal standard normalized matrix factor; IV: Intravenous; LC-MS/MS: Liquid chromatography tandem mass spectrometer; LLE: Liquid-liquid extraction; LLOQ: Lower limit of quantification; MMV: Medicines for Malaria Venture; MRM: Multiple reaction monitoring; MTT: (3-(4, 5-Dimethylthiazol-2-yl)-2, 5-diphenyl tetrazolium bromide; Nom: Nominal; OIS: On-instrument stability; PK: Pharmacokinetic; QC: Quality control; S/N: Signal-to-Noise ratio; SPVS: System performance verification sample; ULOQ: Upper limit of quantification.

## Competing interests

The authors declare that they have no competing interests.

## Authors’ contributions

ETA Developed and validated the LC-MS/MS assay for the quantitative determination of TK900D and TK900E in mouse blood, and used the assay for PK-evaluation of the analytes; performed the data acquisition and interpretation of the results presented in the manuscript; compiled data and presented it in the form as it appears in the manuscript. MT synthesized the compounds and supplied us with in vitro activity data. LG assisted with the evaluation of the PK-properties using PK-summit software. LW, KJS and JHW edited, revised and accepted the manuscript, which is part of ETA’s PhD project. KC revised the manuscript. The final version of the manuscript has been read and accepted by all the authors.
